# Associations of Variability in Metabolic Parameters with Lung Cancer: A Nationwide Population-Based Study

**DOI:** 10.3390/cancers13081982

**Published:** 2021-04-20

**Authors:** In Young Cho, Kyungdo Han, Dong Wook Shin, Mi Hee Cho, Jung Eun Yoo, Jong Ho Cho

**Affiliations:** 1 Department of Family Medicine, Kangbuk Samsung Hospital, Sungkyunkwan University School of Medicine, Seoul 03181, Korea; inyoungs.cho@samsung.com; 2 Department of Statistics and Actuarial Science, Soongsil University, Seoul 06978, Korea; 3 Department of Family Medicine, Samsung Medical Center, Sungkyunkwan University School of Medicine, Seoul 06351, Korea; 4 Center for Clinical Epidemiology, Samsung Advanced Institute of Health Science and Technology (SAIHST), Sungkyunkwan University, Seoul 06351, Korea; 5 Department of Digital Health, Samsung Advanced Institute of Health Science and Technology (SAIHST), Sungkyunkwan University, Seoul 06351, Korea; 6 Samsung C&T Medical Clinic, Kangbuk Samsung Hospital, Sungkyunkwan University School of Medicine, Seoul 03181, Korea; mh76.cho@samsung.com; 7 Department of Family Medicine, Healthcare System Gangnam Center, Seoul National University Hospital, Seoul 06236, Korea; 83259@snuh.org; 8 Department of Thoracic Surgery, Samsung Medical Center, Sungkyunkwan University School of Medicine, Seoul 06351, Korea; jongho9595.cho@samsung.com

**Keywords:** lung cancer, risk factors, glucose, blood pressure, cholesterol, body weight

## Abstract

**Simple Summary:**

Lung cancer is currently the most common cancer worldwide. This study investigates whether visit-to-visit variability in metabolic parameters is associated with lung cancer risk. We found that a high variability in fasting blood glucose, systolic blood pressure, total cholesterol, and body weight were each associated with increased risk of lung cancer. A higher number of high-variability parameters were also associated with increased lung cancer risk. Further research is needed to examine whether reducing variability can lead to decreased lung cancer risk.

**Abstract:**

We investigated whether visit-to-visit variability in metabolic parameters is associated with lung cancer risk. We used nationally representative data from the Korean National Health Insurance System, and 8,011,209 lung-cancer-free subjects who underwent over three health examinations from 2005 to 2010 were followed until 2017. Variability of fasting blood glucose, total cholesterol, systolic blood pressure, and body weight were measured by the variability independent of the mean, assessed by quartiles. There were 44,982 lung cancer events. The hazard ratio (HR) and 95% confidence interval (CI) for lung cancer risk was 1.07 (1.04, 1.10) for fasting blood glucose in the highest quartile, 1.08 (1.05, 1.10) for systolic blood pressure, 1.04 (1.01, 1.07) for weight, and 1.11 (1.08, 1.14) for total cholesterol. When comparing ≥3 vs. 0 high-variability metabolic parameters, the HR for lung cancer was 1.18 (95% CI, 1.14, 1.22). However, while ≥3 high-variability parameters showed an increased lung cancer risk in men (HR 1.26, 95% CI 1.21, 1.31), women did not show increased risk (HR 0.99, 95% CI 0.92, 1.06). High variability in each metabolic parameter, and a higher number of high-variability parameters, were associated with increased lung cancer risk.

## 1. Introduction

Lung cancer is currently the most commonly diagnosed cancer worldwide, and the leading cause of deaths due to cancer, according to global cancer statistics [1]. Tobacco smoking is known as the largest risk factor for lung carcinogenesis, but according to estimates by the International Agency for Research on Cancer, approximately 20% of lung cancer cases occur in never-smokers [1]. Lung cancer in nonsmokers has shown molecular and epigenetic differences from lung cancer in smokers [2], and decreasing smoking rates and an increasing incidence of lung cancer in women have identified the need for further etiologic studies [1,3].

Previous studies have suggested associations between lung cancer risk and metabolic parameters such as diabetes mellitus [4], obesity [5], high blood pressure [6], and plasma lipid levels [7]. Possible mechanisms include oxidative stress and inflammation [8], which may lead to reduced intracellular antioxidants in favor of lung carcinogenesis, and increased reactive oxygen species, which may damage deoxyribonucleic acid (DNA) through oxidation or impaired DNA repair [9]. Insulin resistance, and alterations in insulin-like growth factors [10], and adipokines [11] are also suggested as potential mechanisms for these associations.

On the other hand, metabolic parameters fluctuate over time, and these fluctuations do not occur at random and are consistent within an individual [12,13]. Recent studies have shown that, even after adjusting for mean levels of the metabolic parameters, variability in fasting blood glucose (FBG) [14], weight [15], systolic blood pressure (SBP) [16], and total cholesterol (TC) [17] are independent risk factors for all-cause mortality [14,15,16,17] and cardiovascular events [16,17]. In addition, previous studies have demonstrated an association between variability in metabolic parameters and cancers, such as hepatocellular carcinoma [18,19], and multiple myeloma [20]. Metabolic derangements, inflammatory pathways [18], insulin resistance [21], and shortening of telomeres [22] were suggested as possible underlying mechanisms. Regarding lung cancer, the Iowa Women’s Health Study suggested a positive association between weight variability and lung cancer risk [23]. However, the association became non-significant after adjusting for health risk factors such as smoking, and the study was confined to women. Metabolic risk factors tend to cluster and have been suggested to comprise a syndrome; therefore, their variability may interact in an additive manner to exert a greater impact on health [24]. However, to the best of our knowledge, no studies have examined the additive effects of metabolic parameter variability on lung cancer risk, and the variability of other metabolic parameters besides weight, such as SBP, FBG, and TC levels, have not yet been studied with a focus on lung cancer.

Therefore, our study used a nationwide population-based database to investigate whether the variability of the metabolic parameters FBG, weight, SBP, and TC were associated with increased lung cancer risk, and whether additive effects exist.

## 2. Materials and Methods

### 2.1. Study Population

The Korean National Health Insurance Service (NHIS) provides medical coverage to 97% of the Korean population and medical aid for 3% of the population. The NHIS includes data regarding qualification for insurance (age, sex, income level), diagnosis codes following the International Classification of Disease 10th revision (ICD-10), and information on medical services provided through claims data submitted by healthcare providers [25]. In addition, the NHIS provides health examination programs that include a general exam focused on cardiovascular risk factors for all insured employees, or those over 40 years of age every 2 years [26]. Questionnaires on lifestyle behavior, past medical history, and family history are also recorded. The NHIS database has been used in many epidemiological studies, and details can be found elsewhere [25,26].

**Figure 1:**
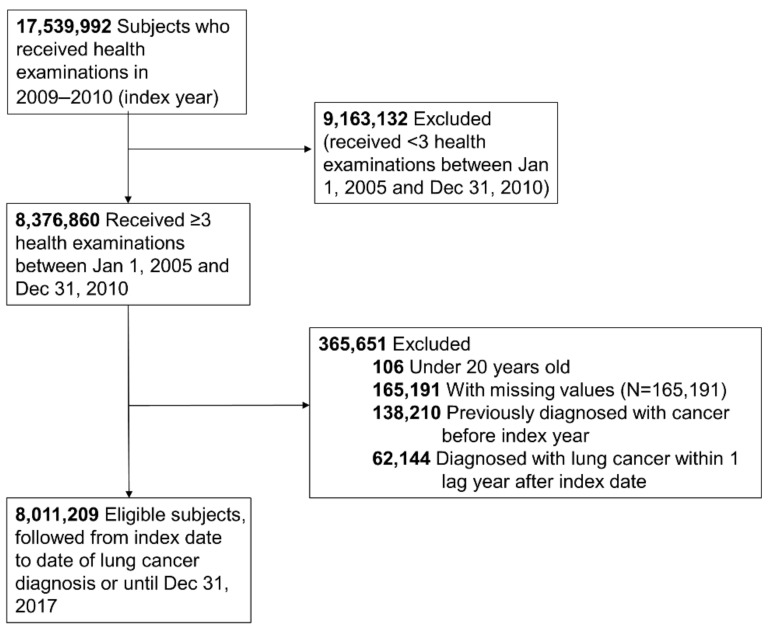
Flowchart of the study population.

In our study, we included those who received a health examination between 2009 and 2010 (index year) and two or more health examinations within the previous 5 years from the index year. Of the 17,539,992 people eligible for a health examination in the index year, 8,376,860 received over three health examinations during the period described. We excluded those under 20 years old, those who had missing data for the variables studied (*n* = 165,191), those with a previous diagnosis of cancer (*n* = 138,210) before the index date, and those diagnosed with lung cancer within 1 year after the index date (*n* = 62,144) for 1 year of lag time. Ultimately, the study population included 8,011,209 subjects (Figure 1). This study was conducted in accordance with the amended Declaration of Helsinki, and received approval by the Institutional Review Board of Samsung Medical Center (IRB No. SMC 2019-07-031), and the need for informed consent was waived because we used deidentified data for our analysis.

### 2.2. Definitions of Variability

Variability was defined as intraindividual variability measured by variability independent of the mean (VIM) in FBG, weight, SBP, and TC values recorded during the health examinations. VIM was calculated by the equation 100 × standard deviation (SD)/mean^β^; β is the regression coefficient, which is the natural logarithm of the SD divided by the natural logarithm of the mean [27]. To analyze whether there was a dose–response association between the aggregate effect of all metabolic parameters and lung cancer risk, we assigned a score of 1 for the highest quartile (Q4) of each metabolic parameter [28]. Subjects were divided into four groups according to the sum of the score assigned for each metabolic parameter: 0, 1, 2, ≥3.

### 2.3. Study Outcomes and Follow-Up

The primary endpoint was the occurrence of lung cancer, defined by recording of the ICD-10 code C34. The study population was followed from baseline to date of new lung cancer diagnosis, death, or until 31 December 2017, whichever came first.

### 2.4. Covariates

Information on current smoking, alcohol consumption, and physical activity were obtained through questionnaires at the index year health examination. Regular physical activity was defined as moderate physical activity for more than 30 min at least 5 times per week, or strenuous physical activity performed for more than 20 min at least 3 times per week.

Diagnosis of diabetes was defined by if subjects had at least one claim for the ICD-10 codes E10–14 per year and prescription of antidiabetic medication, or if FBS level was ≥126 mg/dL at the health examination. Diagnosis of hypertension was defined by if subjects had at least one claim for the ICD-10 codes I10 or I11 per year and a prescription for an antihypertensive medication, or if SBP ≥ 140 mmHg or diastolic blood pressure (DBP) ≥ 90 mmHg at the health examination. Diagnosis of dyslipidemia was defined by if subjects had at least one claim for the ICD-10 code E78 per year and a prescription for a lipid-lowering medication, or if the TC level was ≥240 mg/dL at the health examination.

### 2.5. Statistical Analysis

Comparisons of the baseline characteristics were performed using Pearson’s chi-squared tests and Student’s *t*-tests. We analyzed the hazard ratio (HR) and 95% confidence intervals (CI) for lung cancer using Cox proportional-hazards modeling: model 1 was adjusted for age, sex, smoking, alcohol consumption and physical activity, and model 2 was additionally adjusted for baseline body mass index (BMI), FBG, TC, SBP, and household income. Incidence of lung cancer according to the number of high-variability metabolic parameters was also calculated using Kaplan–Meier curves. Potential effect modification by age group, sex, smoking status, and presence of diabetes, hypertension, or dyslipidemia was evaluated through stratified analyses. As men and women showed different patterns, we further presented the results with each parameter stratified by sex. All statistical analyses were performed using SAS version 9.4 (SAS Institute Inc., Cary, NC, USA), and two-sided *p*-values of <0.05 were considered statistically significant.

## 3. Results

### 3.1. Baseline Characteristics of Study Participants

Subjects with more high-variability parameters were older and more likely to be female, non-smokers, and non-drinkers (Table 1). Baseline mean values, as well as VIM of each parameter of interest (FBG, weight, SBP, TC), increased gradually with the number of high-variability parameters.

### 3.2. Lung Cancer Risk

A total of 44,982 cases of lung cancer occurred during a mean (±SD) follow-up of 6.9 (±0.8) years. For each metabolic parameter, the risk of lung cancer was highest in the highest VIM quartile group, compared with the lowest quartile, even after adjusting for baseline metabolic parameters (Table 2). HR (95% CI) of lung cancer in the highest quartile was 1.06 (1.03, 1.09) for FBG, 1.04 (1.01, 1.06) for weight, 1.07 (1.05, 1.10) for SBP, and 1.11 (1.08, 1.14) for TC.

**Figure 2:**
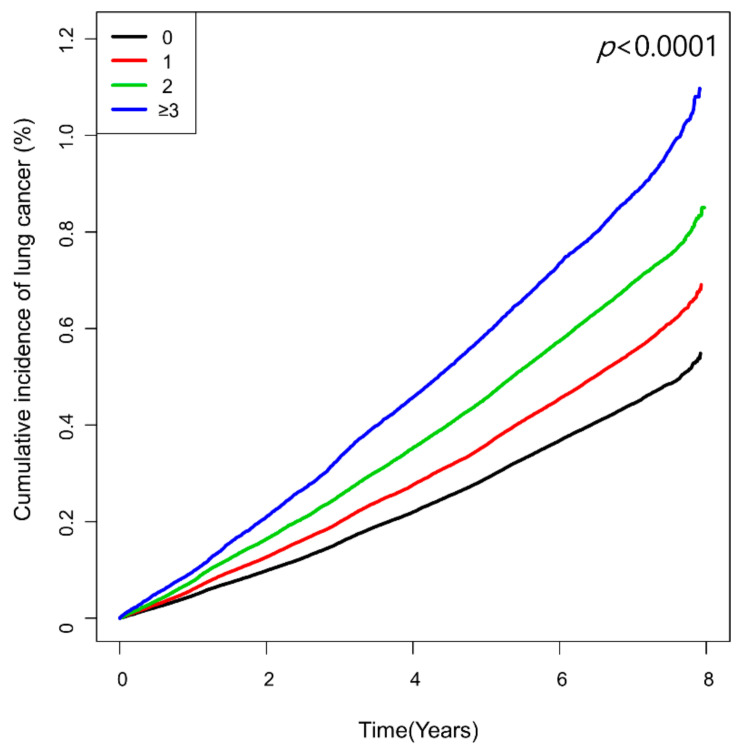
Kaplan–Meier curves showing cumulative incidence of lung cancer according to the number of high-variability parameters defined as the highest quartile of variability independent of the mean (VIM).

The number of high-variability parameters showed a graded association with lung cancer risk (Table 2 and Figure 2). Compared with the reference group of low-variability for all four parameters, the group with ≥3 high-variability parameters had the highest risk of lung cancer (HR 1.18, 95% CI 1.14, 1.22), followed by those with two high-variability parameters (HR 1.13, 95% CI 1.11, 1.16), and one high-variability parameter (HR 1.08, 95% CI 1.06, 1.11).

### 3.3. Subgroup Analyses

**Figure 3:**
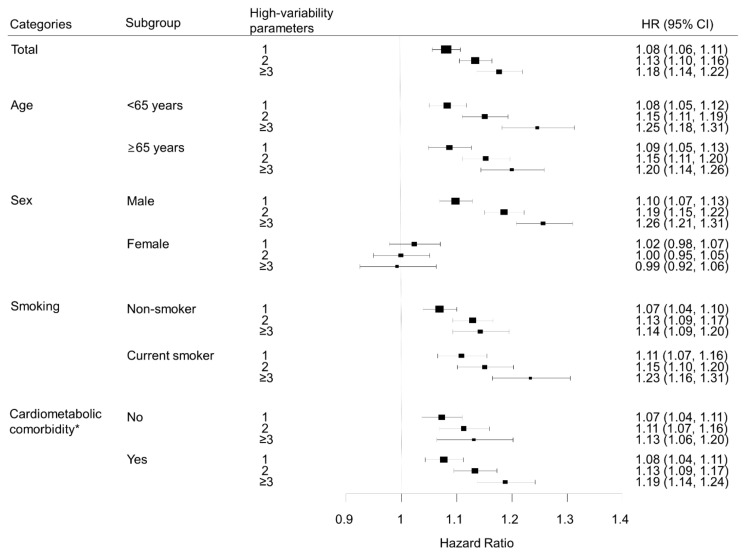
Subgroup analysis of the association between number of high-variability parameters and lung cancer, analyzed by age, sex, smoking status, and cardiometabolic comorbidity. All hazard ratios (HR) are adjusted for age, sex, alcohol consumption, smoking, regular exercise, household income, baseline fasting blood glucose, total cholesterol, systolic blood pressure, and body mass index. * Cardiometabolic comorbidity was defined as the presence of any of hypertension, diabetes mellitus, or dyslipidemia.

**Figure 4:**
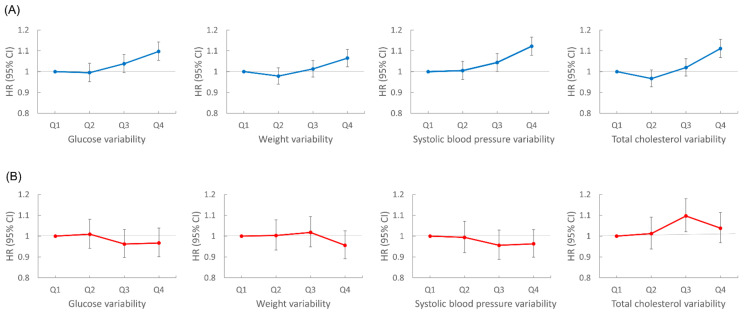
Sex stratified analyses of lung cancer risk according to variability of metabolic parameters. All hazard ratios (HR) are adjusted for age, alcohol consumption, smoking, regular exercise, household income, baseline fasting blood glucose, total cholesterol, systolic blood pressure, and body mass index. (**A**) Risk of lung cancer in men according to glucose, weight, systolic blood pressure, and total cholesterol variability. (**B**) Risk of lung cancer in women according to glucose, weight, systolic blood pressure, and total cholesterol variability.

We performed subgroup analyses according to age group, sex, smoking status, and presence of diabetes, hypertension, or dyslipidemia (Figure 3). Lung cancer risk increased with the number of high-variability parameters in all subgroups, except for women. In sex-stratified analyses, men showed patterns similar to the overall population; for each metabolic parameter, lung cancer risk was highest in the highest VIM quartile group compared with the lowest quartile after adjustment for baseline parameters: HR (95% CI) for FBG in the highest quartile was 1.10 (1.05, 1.14); for weight, 1.07 (1.02, 1.11); for SBP, 1.12 (1.08, 1.17); for TC, 1.11 (1.07, 1.16). However, women did not show significant associations between variability of metabolic parameters and lung cancer risk. In women, for the highest VIM quartile of each metabolic parameter, HR (95% CI) for lung cancer was 0.97 (0.90, 1.04) for FBG, 0.96 (0.89, 1.03) for weight, 0.96 (0.90, 1.03) for SBP, and 1.04 (0.97, 1.11) for TC (Figure 4).

## 4. Discussion

In this nationwide, population-based study, the highest variabilities of FBG, weight, SBP, and TC were associated with a higher risk of lung cancer even after adjustment for variables including baseline FBG, weight, SBP, and TC. Furthermore, our study is the first to suggest that a high variability of FBG, SBP, and TC may be associated with increased lung cancer risk. We were also able to observe a dose-dependent relationship between the number of high-variability parameters and lung cancer risk for the first time.

Our study showed a positive association between weight variability and lung cancer. This is consistent with previous studies, which showed positive associations between weight variability and cancer, such as HCC [18]. In a population-based study, variability in weight was also associated with cancer-related mortality regardless of weight change direction or initial BMI [29]. High weight variability is associated with elevated insulin and shorter telomere length, which may lead to an increased cancer risk [22,30].

Regarding glycemic variability, a previous study on diabetic patients conducted in Japan showed a dose-dependent relationship between development of ‘all’ cancers and high glycemic variability, but not mean hemoglobin A1c (HbA1c) [31]. However, lung cancer was not specifically examined in this study. Compared to high-but-stable glucose levels, oscillating glucose levels have been shown to have a greater impact on oxidative stress generation and endothelial dysfunction [32], which may contribute to carcinogenesis. A previous study that also included lung cancer cases showed that endothelial dysfunction was associated with increased cancer risk [33]. Endothelial dysfunction has been suggested to cause chronic hypoxia, which could decrease deoxyribonucleic acid (DNA) repair and genetic stability [34], and stimulate angiogenesis [35,36].

The exact mechanisms through which the variability of SBP can affect lung cancer risk remain to be elucidated. In a previous cohort study, high BP was associated with increased lung cancer [6]; abnormalities in the proliferation of vascular smooth muscle cells associated with hypertension were suggested to be associated with carcinogenesis [37] through abnormal apoptotic function [38] or shortened telomeres [39]. Meanwhile, BP variability and hemodynamic instability have been suggested to cause oxidative stress, inflammation, and endothelial dysfunction [40]. Oxidative stress may be linked to increased lung cancer risk through inflammation, DNA damage, inhibition of apoptosis, activation of carcinogenesis through signal transduction pathways, and lipid peroxidation [41,42,43]. Meanwhile, inflammation is expected to increase lung cancer risk by promoting antiapoptotic signals, leading to angiogenesis and providing oxygen and nutrients to tumor cells, allowing them to grow [44,45].

Our study is the first to show an association between TC variability and lung cancer risk. While the mechanism underlying this association is unclear, a meta-analysis found an inverse association between TC and lung cancer risk; disturbance of cholesterol metabolism was suggested to be an underlying mechanism [7]. Regarding lipid variability, a recent study found that high-density lipoprotein-cholesterol variability was associated with multiple myeloma [20]. The variability of cholesterol levels was suggested to contribute to carcinogenesis through a shared common inflammation process, and modification of gene expression in cancer cells [20]. Further research is warranted to clarify the mechanism for the association between TC variability and lung cancer.

Because lung cancer risk increased in a dose-dependent pattern along with an increasing number of high-variability parameters, the associations of each parameter’s variability may be additive. Furthermore, lung cancer risk was greatest in the presence of three to four high-variability metabolic parameters compared with any single parameter. Variability of blood pressure, cholesterol, or glucose levels may have been caused by non-adherence to treatment for hypertension, dyslipidemia, or diabetes [46,47], which may have also been associated with negative health behaviors such as smoking [48,49]. However, stratified analyses according to cardiometabolic comorbidity and smoking status showed that the association between the number of high-variability metabolic parameters and lung cancer risk was consistent regardless of these cardiometabolic comorbidities.

The variability of metabolic parameters was not associated with lung cancer risk in the subgroup analysis of women, possibly due to differences in lung cancer histology or metabolic pathophysiology between men and women. More female lung cancer patients are histologically diagnosed with adenocarcinoma [50], and lung cancer subtypes may be differently affected by metabolic parameters, as shown by a study where lung adenocarcinoma displayed significant glucose independence compared to squamous cell carcinoma [51]. There may also be genetic reasons, such as a higher frequency of epidermal growth factor receptor (EGFR) mutations found in women [52]. EGFR mutant lung cancer cells were shown to be more dependent on cholesterol for proliferation compared to EGFR wild-type cancer cells [53]; it could be speculated that EGFR mutation plays a role between lipid metabolism and lung cancer carcinogenesis. However, further research is needed to reveal the relationship between EGFR and metabolic variability. Differences in sex hormones, metabolic regulation, body fat composition, lipid metabolism, and insulin resistance between men and women may also lead to differences in lung cancer risk associated with metabolic variability [54]. A previous study also raised the possibility that lung cancer may grow more slowly in women than men; therefore, the follow-up time in our study may have been insufficient to observe a significant effect in women [3,55]. Further research on the different associations between metabolic parameters and lung cancer in males and females is warranted.

### Clinical Implications

The variability of metabolic parameters may be a useful target for intervention for preventive methods against lung cancer. For example, reducing blood pressure variability may be an important target in hypertensive high-risk patients. Since previous studies have shown that calcium-channel blockers (CCBs) are the most effective antihypertensive for reducing blood pressure variability [56], prescribing CCBs as the treatment of choice may be helpful for high-risk patients. Glucose variability is also greater in diabetic patients with poor glycemic control, and so emphasis on medication adherence and diet quality [57], as well as the use of antidiabetic agents known to lower glycemic variability such as a glucagon-like peptide-1 agonist or sodium-glucose cotransporter 2 [58,59], may be useful for high-risk patients. Meanwhile, statins used at higher dosages are associated with decreased lipid variability [60] and, although there are conflicting data [61,62], recent studies have suggested that statins may have a protective effect against lung cancer risk [63], suggesting an additional benefit for high-risk patients. A multidisciplinary approach that includes lifestyle modification, such as adequate physical activity and diet, may also have beneficial effects on metabolic parameters and variability. For instance, a multidisciplinary intervention for metabolic syndrome including physical activity training and diet was shown to improve both blood pressure variability and HbA1c levels [64].

Despite the strengths of our study, including a large nationwide database, there are some limitations to be mentioned. First, this was an observational study, and the observed associations may not be causal. To minimize the effects of reverse causality, we excluded those diagnosed with lung cancer within one year of the index date. Second, unknown factors that may influence the variability of metabolic parameters may have also influenced lung cancer risk. Third, we did not have information on the histologic type of lung cancer in the claims data. Fourth, we could not determine whether body weight changes were unintentional. Finally, our study was based on Korean data, and therefore the results may not be generalizable to other ethnic populations.

## 5. Conclusions

High variability in metabolic parameters may be associated with increased lung cancer risk. A higher number of high-variability parameters was associated with a higher lung cancer risk in a dose-dependent manner, and the results were consistent in diverse subgroups, except for women. Further research may help confirm these results, explore the mechanisms, and examine whether interventions that target reducing metabolic variability can lead to decreased lung cancer risk.

## Figures and Tables

**Table 1:** Baseline characteristics of study population by metabolic parameter variability index.

	Metabolic Parameter Variability Index				
N	0	1	2	≥3	*p*-Value
	2,717,080	3,143,249	1,638,357	512,523	
Age (years)	47.0 ± 12.6	47.8 ± 13.6	49.1 ± 14.6	50.9 ± 15.5	<0.0001
Sex (male)	1,713,623 (63.1)	1,838,363 (58.5)	899,735 (54.9)	265,490 (51.8)	<0.0001
Smoking					<0.0001
Current, ≥20 cigarettes/d	300,149 (11.2)	357,894 (11.5)	187,225 (11.5)	57,279 (11.3)	
Current, 10–19 cigarettes/d	309,947 (11.6)	350,470 (11.3)	172,483 (10.6)	49,882 (9.8)	
Current, <10 cigarettes/d	73,226 (2.7)	82,665 (2.7)	42,841 (2.6)	13,481 (2.7)	
Ex-smoker, ≥20 cigarettes/d	197,683 (7.4)	220,694 (7.1)	115,329 (7.1)	36,941 (7.3)	
Ex-smoker, 10–19 cigarettes/d	202,687 (7.6)	199,952 (6.4)	92,182 (5.7)	25,932 (5.1)	
Ex-smoker, <10 cigarettes/d	66,945 (2.5)	67,013 (2.2)	31,226 (1.9)	9,217 (1.8)	
Non-smoker	1,524,265 (57.0)	1,827,619 (58.8)	980,858 (60.5)	315,241 (62.1)	
Alcohol consumption					<0.0001
None	1,275,385 (46.9)	1,585,324 (50.4)	881,290 (53.8)	295,313 (57.6)	
Mild to moderate (<30 g/day)	1,228,722 (45.2)	1,316,605 (41.9)	632,673 (38.6)	178,623 (34.9)	
Heavy (≥30 g/day)	212,973 (7.8)	241,320 (7.7)	124,394 (7.6)	38,587 (7.5)	
Regular physical activity	554,176 (20.4)	618,857 (19.7)	310,185 (18.9)	91,513 (17.9)	<0.0001
Household income					<0.0001
Q1	497,420 (18.3)	656,015 (20.7)	376,498 (23.0)	124,962 (24.4)	
Q2	678,140 (25.0)	857,948 (27.3)	469,876 (28.7)	149,408 (29.2)	
Q3	782,055 (28.8)	890,502 (28.3)	449,887 (27.5)	137,954 (26.9)	
Q4	759,465 (28.0)	738,784 (23.5)	342,096 (20.9)	100,199 (19.6)	
Diabetes, yes	132,900 (4.9)	251,351 (8.0)	197,392 (12.1)	92,535 (18.1)	<0.0001
Hypertension, yes	577,053 (21.2)	809,433 (25.8)	506,657 (30.9)	189,502 (37.0)	<0.0001
Dyslipidemia, yes	307,439 (11.3)	489,095 (15.7)	332,534 (20.3)	130,019 (25.4)	<0.0001
Chronic kidney disease, yes	162,774 (6.0)	195,852 (6.2)	116,477 (7.1)	44,695 (8.7)	<0.0001
Metabolic syndrome, yes					
Weight (kg)	65.1 ± 11.2	64.5 ± 11.5	63.9 ± 11.8	63.1 ± 12.1	<0.0001
Height (cm)	165.4 ± 8.9	164.4 ± 9.2	163.4 ± 9.3	162.4 ± 9.5	<0.0001
Waist circumference (cm)	80.4 ± 8.7	80.5 ± 8.9	80.7 ± 9.0	81.0 ± 9.2	<0.0001
Body mass index (kg/m^2^)	23.7 ± 3.0	23.8 ± 3.1	23.8 ± 3.3	23.8 ± 3.4	<0.0001
Fasting blood glucose (mg/dL)	95.3 ± 16.9	96.7 ± 21.1	98.6 ± 25.5	101.3 ± 30.8	<0.0001
Systolic BP (mmHg)	122.4 ± 13.0	122.4 ± 14.5	122.6 ± 15.9	122.8 ± 17.4	<0.0001
Diastolic BP (mmHg)	76.6 ± 9.3	76.4 ± 9.8	76.3 ± 10.2	76.3 ± 10.7	<0.0001
Total cholesterol (mg/dL)	196.4 ± 33.2	195.7 ± 35.8	195.3 ± 38.9	194.9 ± 42.7	<0.0001
HDL cholesterol (mg/dL)	54.8 ± 19.1	55.1 ± 19.8	55.3 ± 20.7	55.5 ± 21.9	<0.0001
LDL cholesterol (mg/dL)	116.6 ± 44.5	115.2 ± 46.2	114.1 ± 5	112.7 ± 49.0	<0.0001
Triglycerides (geometric mean)	112.5 (112.4–112.5)	113.6 (113.5–113.7)	115.7 (115.6–115.8)	118.7 (118.5–118.9)	<0.0001
Glucose VIM	7.12 ± 3.07	9.9 ± 5.7	12.5 ± 6.6	15.7 ± 6.5	<0.0001
Weight VIM	1.3 ± 0.6	1.9 ± 1.3	2.2 ± 1.6	3.3 ± 1.9	<0.0001
Systolic BP VIM	6.9 ± 2.9	9.3 ± 4.9	11.4 ± 5.5	13.9 ± 5.3	<0.0001
Total cholesterol VIM	13.8 ± 5.6	18.7 ± 10.7	24.6 ± 12.9	31.5 ± 13.1	<0.0001

Abbreviations: N, number; Q, quartile; BP, blood pressure; VIM, variability independent of the mean. Metabolic variability index was defined as the number of high-variability metabolic parameters (fasting blood glucose, body weight, systolic blood pressure, and total cholesterol levels). *p*-values were calculated using the Chi square test for categorical variables and Student’s *t*-test or the Mann–Whitney U test for continuous variables.

**Table 2:** Lung cancer risk by quartiles of metabolic parameter variability and number of high-variability metabolic parameters.

	N	Events (*n*)	Follow-Up Duration (Person-Years)	Incidence Rate per 1000	HR (95% CI) Model 1 ^1^	HR (95% CI) Model 2 ^2^
**Glucose variability** (**VIM**)						
Q1	2,002,582	10,692	13,733,341	0.78	1 (Reference)	1 (Reference)
Q2	2,003,021	10,577	13,852,596	0.76	1.03 (1.00, 1.06)	1.03 (1.00, 1.06)
Q3	2,002,771	10,799	13,890,020	0.78	1.04 (1.01, 1.07)	1.03 (1.01, 1.06)
Q4	2,002,835	12,914	13,846,359	0.93	1.08 (1.05, 1.10)	1.06 (1.03, 1.09)
*p* for trend					<0.001	<0.001
**Weight variability** (**VIM**)						
Q1	2,003,177	11,248	13,828,884	0.81	1 (Reference)	1 (Reference)
Q2	2,002,517	10,659	13,903,250	0.77	0.99 (0.97, 1.02)	0.99 (0.96, 1.01)
Q3	2,003,817	11,088	13,877,666	0.80	1.03 (1.01, 1.06)	1.02 (0.99, 1.05)
Q4	2,001,698	11,987	13,712,516	0.87	1.06 (1.04, 1.09)	1.04 (1.01, 1.06)
*p* for trend					<0.001	0.001
**Systolic blood pressure variability** (**VIM**)						
Q1	2,034,696	10,478	14,014,465	0.75	1 (Reference)	1 (Reference)
Q2	1,970,945	9321	13,675,543	0.68	1.00 (0.97, 1.03)	1.00 (0.97, 1.02)
Q3	1,998,818	10,952	13,875,173	0.79	1.03 (1.00, 1.06)	1.02 (1.00, 1.05)
Q4	2,006,750	14,231	13,757,135	1.03	1.09 (1.06, 1.11)	1.07 (1.05, 1.10)
*P* for trend					<0.001	<0.001
**Total cholesterol variability** (**VIM**)						
Q1	2,002,798	10,232	13,794,745	0.74	1 (Reference)	1 (Reference)
Q2	2,002,798	9683	13,915,420	0.70	0.99 (0.96, 1.01)	0.98 (0.96, 1.012)
Q3	2,002,811	10,692	13,889,463	0.77	1.04 (1.01, 1.07)	1.03 (1.01, 1.062)
Q4	2,002,802	14,375	13,722,688	1.05	1.11 (1.09, 1.14)	1.11 (1.08, 1.136)
*p* for trend					<0.001	<0.001
**Number of high-variability metabolic parameters**						
0	2,717,080	12,049	18,872,698	0.64	1 (Reference)	1 (Reference)
1	3,143,249	17,319	21,720,541	0.80	1.09 (1.07, 1.12)	1.08 (1.06, 1.11)
2	1,638,357	11,193	11,248,022	1.00	1.16 (1.16, 1.19)	1.13 (1.11, 1.16)
≥3	512,523	4421	3,481,056	1.27	1.21 (1.17, 1.25)	1.18 (1.14, 1.22)
*p* for trend					<0.001	<0.001

Abbreviations: N, number of subjects; *n*, number of lung cancer events; HR, hazard ratio; Q, quartile; VIM, variability independent of the mean. ^1^ Model 1: adjusted for age, sex, smoking, alcohol consumption, regular physical activity. ^2^ Model 2: adjusted for variables in model 1, household income, baseline body mass index, baseline fasting blood glucose, baseline cholesterol, and baseline systolic blood pressure.

## Data Availability

Restrictions apply to the availability of these data. Data was obtained from the Korean National Health Insurance Sharing Service and are available from the authors with the permission of the Korean National Health Insurance Sharing Service.
